# A new inland centre for Dicranomyia (Erostrata) (Diptera, Limoniidae): first record of the subgenus in mainland China, with three species including one new species

**DOI:** 10.3897/zookeys.1296.201582

**Published:** 2026-11-06

**Authors:** Liying Dai, Juan Chen, Shuo Ma, Pengxuan Guo, Xiao Zhang

**Affiliations:** 1 Shandong Engineering Research Centre for Environment-Friendly Agricultural Pest Management, College of Plant Health and Medicine, Qingdao Agricultural University, Qingdao 266109, China Shandong Engineering Research Centre for Environment-Friendly Agricultural Pest Management, College of Plant Health and Medicine, Qingdao Agricultural University Qingdao China

**Keywords:** Biogeography, key, new records, Tipuloidea

## Abstract

The subgenus Dicranomyia (Erostrata) Savchenko, 1976 is recorded from mainland China for the first time with the documentation of three species. One species new to science, D. (E.) serrata**sp. nov**., and two species newly recorded from China, D. (E.) globulithorax Alexander, 1924 and D. (E.) koreana Kim & Bae, 2023, are reported. With these additions, the number of confirmed *Erostrata* species in China is increased from one to four. Detailed descriptions, photographs, and a key to the *D.* (*Erostrata*) species worldwide are provided. The updated distribution reveals China as an emerging inland centre of diversity for the subgenus, bridging the coastal East Asian core with the Himalayan and Southeast Asian peripheries.

## Introduction

The subgenus Dicranomyia (Erostrata) was established by Savchenko in Savchenko and Krivolutskaya (1976) with D. (E.) globithorax Osten Sacken, 1869 as the type species. The subgenus is characterised by the following diagnostic features: body medium sized; antennae short, if bent backwards, ending before wing base, flagellum with proximal segments short-pedicellate or subglobose; rostrum and palpus very short, reduced; pleuron smooth; mesothorax gibbous; wing translucent, unpatterned; sternite 7 of male abdomen usually with an internal sac at the posterior margin (except D. (E.) reniformis Kato, Tachi & Gelhaus, 2018 and D. (E.) yazuensis Kato, Tachi & Gelhaus, 2018, which have only a darkened area or notch and no internal sac); tergite 9 with narrow apex, bearing a small angular notch, ventromesal lobe of gonocoxite simple; outer gonostylus missing, inner gonostylus with or without rostral prolongation; rostral spines (usually two in number, but one in D. (E.) yazuensis Kato, Tachi & Gelhaus, 2018) shifted to the lateral surface of gonostyli; gonostyli either with dense brush of rigid spiniform setae along mesal margin, or with a dorsal row of such setae before apex, though some species (e.g. D. (E.) jejuensis Kim & Bae, 2023, D. (E.) koreana Kim & Bae, 2023, D. (E.) reniformis Kato, Tachi & Gelhaus, 2018, D. (E.) tabashii (Alexander, 1934), and D. (E.) yazuensis Kato, Tachi & Gelhaus, 2018) instead bear a spine or a lobe at the gonostylus apex; aedeagus distinctly bifurcate at apex, sometimes deeply split (Savchenko and Krivolutskaya 1976; Podenas et al. 2019). All species of *D.* (*Erostrata*) for which ecological data are available are associated with humid, mature forests with an abundance of decaying wood and fungal growth. Dicranomyia (E.) globithorax has been reared from “sodden, fungus-riddled hardwood logs” in North America (Rogers 1942); in Korea, D. (E.) tabashii occurs along mountainous streams with lots of fallen leaves and moss, and D. (E.) koreana in wet deciduous forests by intermittent streams (Kim and Bae 2023).

The taxonomic history of species, currently belonging to subgenus *Erostrata* dates to the 19^th^ century. Osten Sacken (1869) described *D. globithorax* from Japan; Edwards (1919) added *D. striopleura* (Edwards, 1919) from Sumatra. Between 1924 and 1967, Alexander (1924, 1931a, 1931b, 1934, 1955, 1959, 1967) described numerous species from China, India, Japan, Nepal, the Philippines, and South Korea, which established the basic framework of the group. After the formal erection of the subgenus by Savchenko in Savchenko and Krivolutskaya (1976), no further *D.* (*Erostrata*) species were described for more than 40 years, until Kato et al. (2018) revised the Japanese fauna, describing three new species and raising two subspecies of D. (E.) globithorax to species rank. Podenas et al. (2019, 2020) subsequently studied the Korean species, and Kim and Bae (2023) added two more Korean species and provided the *COI* barcode-based molecular phylogeny of the subgenus. Their neighbour-joining tree divided *D.* (*Erostrata*) into two deep clades corresponding to the presence or absence of numerous strong black spines on the mesal face of gonostylus, and they suggested that these clades might even warrant subgeneric status.

Prior to this study, 14 species were recognised worldwide: eight in the Palaearctic Region, six in the Oriental Region, and one in the Nearctic Region (Oosterbroek 2026). Most species are concentrated in Japan, the Korean Peninsula, and the Russian Far East, with a few extending to the Philippines, Indonesia, Malaysia, and the southern slopes of the Himalayas. However, China remained a near-blank spot on the global distribution map: only D. (E.) melas (Alexander, 1934) was recorded from Taiwan, and no species had ever been recorded from mainland China. Given the continuity of temperate–subtropical forest ecosystems between eastern China, Korea, and Japan, and the geological links between southwestern China and the Himalayas, this absence clearly reflected insufficient sampling rather than a true biogeographical vacuum.

Based on general entomological surveys conducted in several provinces of mainland China, the present study provides the first records of *D.* (*Erostrata*) from mainland China. The Chinese fauna is here taxonomically revised and includes three species: D. (E.) serrata sp. nov. from Sichuan is described as new to science, and D. (E.) globulithorax Alexander, 1924 and D. (E.) koreana Kim & Bae, 2023 are recorded from China for the first time. Through an extensive literature review and the examination of specimens, an identification key to species worldwide, revised and updated from that of Kim and Bae (2023), is provided here, and the biogeographical implications of the updated distribution are discussed.

## Material and methods

### Specimen collection and preservation

The examined material was derived mainly from specimens deposited in the Insect Taxonomy Laboratory of Qingdao Agricultural University and its partner institutions. All adult specimens were collected by sweep netting and in Malaise traps. Both previously archived and newly collected individuals were preserved in 95% ethanol and subsequently stored at –20 °C in the collection facility of Qingdao Agricultural University (**QAU**) and China Agricultural University (**CAU**). Detailed collection data for each species are provided under the respective species accounts.

### Morphological study

External morphology was examined under a Phenix SMZ180-LB stereomicroscope. To allow clearer observation of the structural details, male terminalia were macerated in 10% NaOH at room temperature for 12–15 h, rinsed in distilled water, and examined in glycerol. Photographs were taken using Canon EOS 5D and 90D and assembled into plates using Photoshop. Morphological terminology follows Cumming and Wood (2017) and de Jong (2017).

## Results

### Taxonomy

Key to species of Dicranomyia (Erostrata) worldwide, based on males

**Table:** 

1	Tarsi white (Alexander 1967)	**D. (E.) congesta (Alexander, 1967)**
–	Tarsi not white	**2**
2	Pleuron with broad, blackish-brown lateral stripe (Kim and Bae 2023)	**D. (E.) striopleura (Edwards, 1919)**
–	Pleura without this feature	**3**
3	Claw long and slender, with spines at only extreme base; wing relatively broad (Alexander 1959)	**D. (E.) cnephosa (Alexander, 1959)**
–	Claw not as above; wing normal in size	**4**
4	Mesal face of gonostylus without numerous strong, black spines	**5**
–	Mesal face of gonostylus with numerous strong, black spines	**9**
5	Prescutum with a dark longitudinal stripe; gonostylus reniform (Kato et al. 2018: fig. 14)	**D. (E.) reniformis Kato, Tachi & Gelhaus, 2018**
–	Prescutum without stripe; gonostylus not as above	**6**
6	Gonostylus with a small protuberance on outer dorsal surface bearing one stout seta (Kato et al. 2018: fig. 29)	**D. (E.) yazuensis Kato, Tachi & Gelhaus, 2018**
–	Gonostylus with a small protuberance on outer dorsal surface bearing two stout setae	**7**
7	Sternite 7 of male abdomen with shallow, V-shaped notch with darkened margin; paramere with hooked tip and subapical projection (Kim and Bae 2023: fig. 1D, F, G)	**D. (E.) jejuensis Kim & Bae, 2023**
–	Sternite 7 of male abdomen without the above features; paramere without subapical projection	**8**
8	Sternite 7 of male abdomen with deep, internal, U-shaped sac (Podenas et al. 2019: fig. 125); halter brown with stem obscure yellow (Alexander 1934)	**D. (E.) tabashii (Alexander, 1934)**
–	Sternite 7 with central deep, conical, slightly darkened sac, and rounded entrance (Fig. 5C; Kim and Bae 2023: fig. 3D); halter pale brownish yellow with base and knob darker (Fig. 4D)	**D. (E.) koreana Kim & Bae, 2023**
9	Gonostylus long, triangular	**10**
–	Gonostylus not as above	**11**
10	Ventromesal lobe of gonocoxite very long and slender; gonostylus with spinous setae on mesal face restricted to distal 1/2 (Alexander 1931a: fig. 28)	**D. (E.) canis (Alexander, 1931)**
–	Ventromesal lobe of gonocoxite relatively small; gonostylus densely covered with erect black setae on mesal face (Alexander 1931b: fig. 32)	**D. (E.) cynotis (Alexander, 1931)**
11	Gonostylus tip distinctly concave, forming a long spine and a circular protuberance densely covered with black spines (Fig. 9A, E, F)	**D. (E.) serrata sp. nov**.
–	Gonostylus without this feature	**12**
12	Paramere with special features: black spot or ridge-like, short extension	**13**
–	Paramere simple, without special features	**14**
13	Ventromesal lobe of gonocoxite rounded at tip; paramere with apex produced into a long, curved, black spine and with a small black spot at base of narrow portion (Alexander 1934: fig. 33)	**D. (E.) melas (Alexander, 1934)**
–	Ventromesal lobe of gonocoxite truncated at tip; paramere with apex pointed, dorsomedially curved, bearing a short, ridge-like extension near base of dorsal margin and with lateral part strongly expanded into a ribbon-like plate (Kato et al. 2018: figs 20, 22)	**D. (E.) submelas Kato, Tachi & Gelhaus, 2018**
14	Thorax strikingly gibbous, rising abruptly over head (Osten Sacken 1869); tip of gonostylus slightly arched (Podenas et al. 2019: fig. 115)	**D. (E.) globithorax Osten Sacken, 1869**
–	Thorax less gibbous (Alexander 1924); tip of gonostylus with a postero-lateral margin produced into a distinct angle (Fig. 2A, F)	**D. (E.) globulithorax Alexander, 1924**

#### Dicranomyia (Erostrata) globulithorax

Taxon classificationAnimaliaDipteraLimoniidae

Alexander, 1924

2ADEF7B0-E011-59CA-A258-E16B7FAC8AA6

Figs 1, 2, 3

Dicranomyia globulithorax Alexander, 1924: 547. Type locality: Japan: Hokkaido.
Limonia (Limonia) globithorax globulithorax —Alexander 1955: 278.
Dicranomyia (Erostrata) globithorax globulithorax —Savchenko and Krivolutskaya 1976: 132; Savchenko 1983: 138; Savchenko 1989: 307; Nakamura et al. 2014: 41.
Dicranomyia (Erostrata) globulithorax —Kato et al. 2018: 184.

##### Specimens examined.

China • 1♂; Yunnan Province, Xishuangbanna Dai Autonomous Prefecture; 22°0'36.57"N, 100°47'45.14"E; 2016; Malaise trap; CAU.

**Figure 1.**
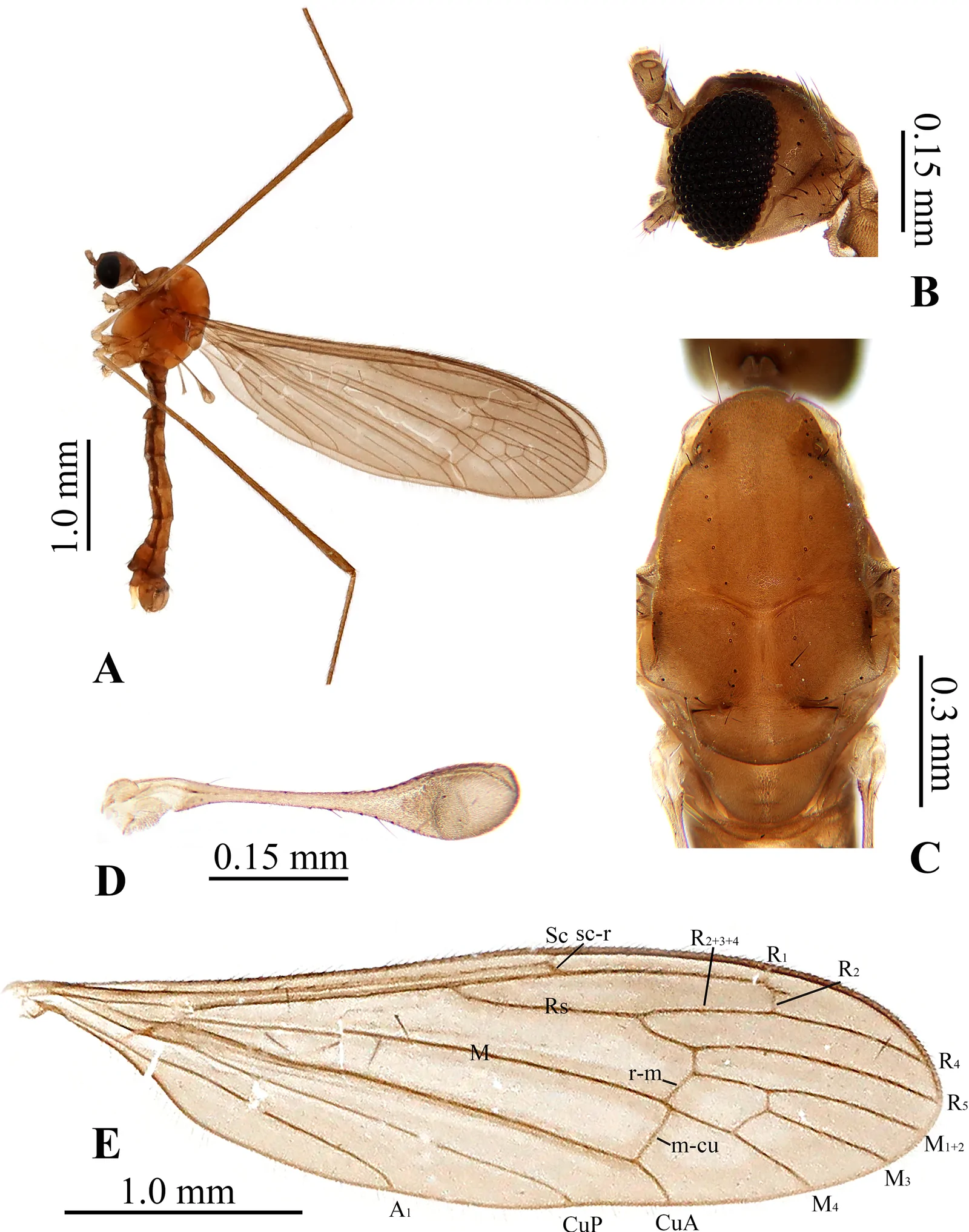
Dicranomyia (Erostrata) globulithorax Alexander, 1924. **A**. Habitus of male, lateral view; **B**. Head, lateral view; **C**. Thorax, dorsal view; **D**. Halter; **E**. Wing.

**Figure 2.**
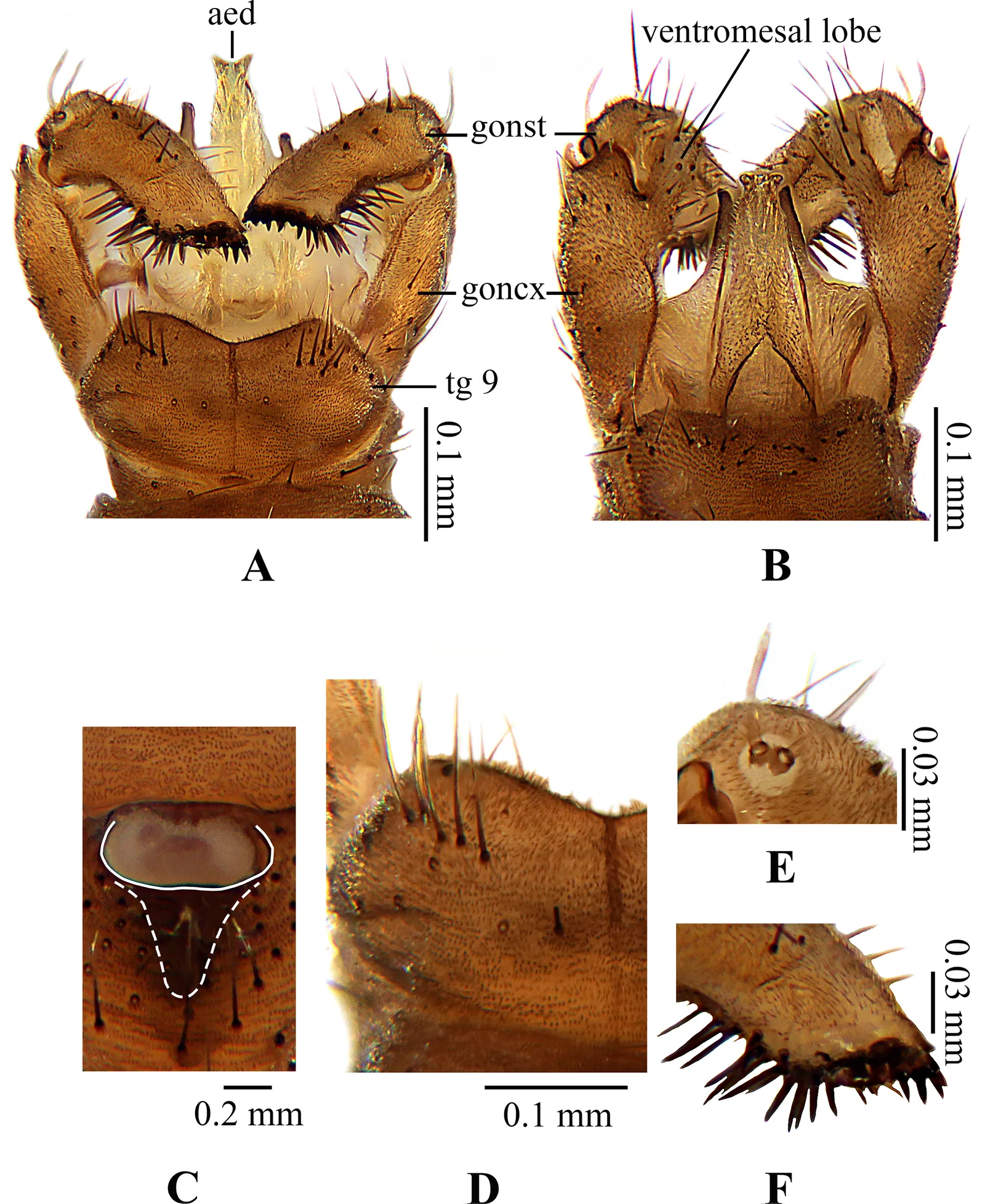
Dicranomyia (Erostrata) globulithorax Alexander, 1924. **A**. Male hypopygium, dorsal view; **B**. Male hypopygium, ventral view; **C**. Sac of male sternite 7, ventral view (outlined boundary); **D**. Tergite 9, dorsal view; **E**. Basal part of gonostylus, lateral view; **F**. Apical part of gonostylus, dorsal view. Abbreviations used in figures: aed = aedeagus; goncx = gonocoxite; gonst = gonostylus; tg = tergite.

**Figure 3.**
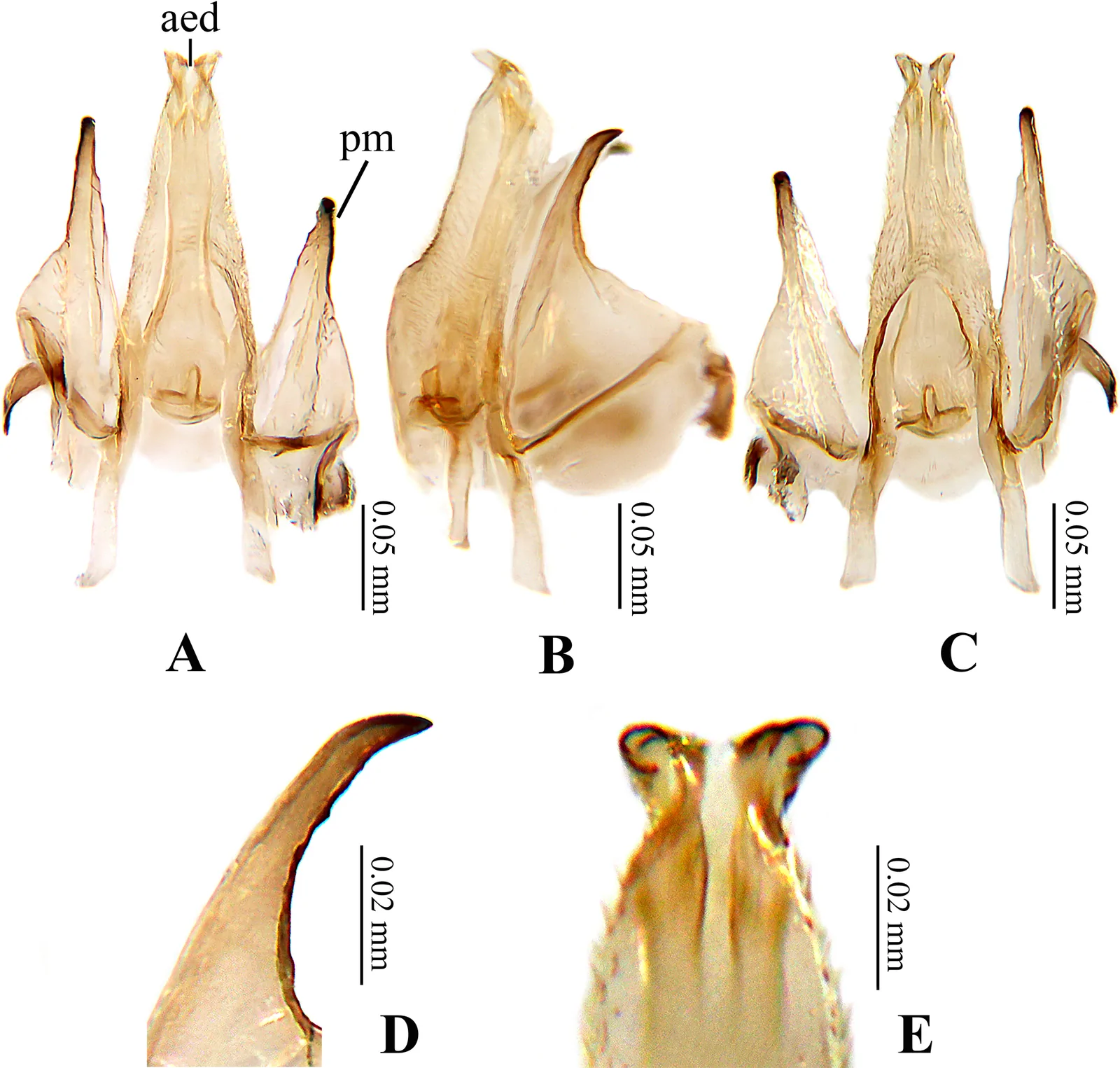
Dicranomyia (Erostrata) globulithorax Alexander, 1924. **A**. Complex of aedeagus, dorsal view; **B**. Complex of aedeagus, lateral view; **C**. Complex of aedeagus, ventral view; **D**. Apical part of paramere, lateral view; **E**. Apical part of aedeagus, ventral view. Abbreviations used in figures: aed = aedeagus; pm = paramere.

##### Diagnosis.

Prescutum and presutural scutum brownish yellow with anterior 1/3 margin slightly darker. Postsutural scutum brown with median area slightly paler. Pleuron brown, darker near base of wing. Wing with slightly darker areas along veins. Vein Sc ending opposite about 1/2 length of Rs; m-cu just beyond base of dm. Tergite 9 posterior margin with a broad and distinct notch. Gonostylus shallowly concave at basal third and tip, tip nearly truncate, bearing dense strong black spines, extending to about midlength of inner side. Paramere tip acute. Aedeagus tip forked and recurved, subtip constricted.

##### Description.

**Male**. Body length 3.0 mm, wing length 4.0 mm.

Head (Fig. 1A, B). Dark brown. Setae on head black. Antennae with scape and pedicel brown. Scape cylindrical, 1.5 times as long as wide. Pedicel oval, 2.0 times as long as wide, tip enlarged. Rostrum short, pale brown. Palpus dark brown, one segment with brown setae observed (additional segments likely absent due to specimen condition).

Thorax (Fig. 1C). Pronotum brownish yellow. Prescutum and presutural scutum brownish yellow with anterior 1/3 margin slightly darker. Postsutural scutum brown with median area slightly paler. Scutellum brownish yellow. Mediotergite brownish yellow. Pleuron (Fig. 1A) brown, darker near base of wing. Thorax with dark brown setae. Coxae (Fig. 1A) pale brown; trochanters slightly paler in comparison to coxae; femora brown, with base slightly paler; tibiae brown. Setae on legs dark brown. Wing (Fig. 1E) brownish yellow, with slightly darker areas along veins; veins brown. Venation: Sc ending opposite about 1/2 length of Rs; sc-r close to tip of Sc; Rs arising from middle of wing, base slightly curved, nearly straight; cell dm closed, 1.5 times as long as wide; m-cu just beyond base of dm. Halter (Fig. 1D) pale brown, knob darker.

Abdomen (Fig. 1A). Segments 1–8 dark brown, segment 8 with posterior margin darker; sternite 7 (Fig. 2C) with an internal sac, broad basally and slightly narrowed to a bluntly rounded apex, bearing an oval opening. Setae on abdomen brown.

Hypopygium (Figs 2, 3). Generally brownish yellow. Tergite 9 (Fig. 2A, D) nearly trapeziform, posterior margin with a broad and distinct notch, each lobe with about 11–13 long brown setae, and median area also with 1–3 setae. Gonocoxite (Fig. 2A, B) cylindrical in dorsal view and fusiform in ventral view; a ventromesal lobe at its apical third, with length about 2/5 of gonocoxite. Gonostylus (Fig. 2A, B, E, F) nearly as long as gonocoxite, outer side at base with two long, stout, transparent setae (Fig. 2E); base of gonostylus broad, surface setose; tip nearly truncate, with a postero-lateral margin produced into a distinct angle, bearing dense strong black spines on about distal 1/2 of inner side (Fig. 2F). Paramere (Fig. 3A–D) in lateral view, broad at base, narrowed and curved outward apically, tip acute. Aedeagus (Fig. 3A–C, E) relatively short, extending to about tip of gonocoxite, widened at base, tip forked and recurved, subtip constricted.

**Female**. Unknown.

##### Distribution.

China (Yunnan); Japan, Russia, South Korea.

##### Remarks.

Dicranomyia (E.) globulithorax was originally described by Alexander (1924) as *Dicranomyia globulithorax* from a male collected at Shikotsu, Hokkaido, Japan, with a detailed description and illustrations of the male genitalia. Alexander (1955) later referred to it as Limonia (Limonia) globithorax globulithorax, adding Japanese distribution records. Savchenko and Krivolutskaya (1976) subsequently recorded it as a subspecies, D. (E.) globithorax globulithorax, from Russia, and with further records added by Savchenko (1983). Kato et al. (2018) elevated it to species rank, providing a redescription and new illustrations of the gonostyli. Podenas et al. (2019) studied Korean populations, providing photographs of antennae, wings, and male terminalia. The specimen from Yunnan discussed here matches the original description and illustration of the male hypopygium, particularly the gonostylus, which is broad, truncate, and densely covered with stout black spiniform setae. This confirms the presence of D. (E.) globulithorax in China for the first time, representing a considerable south-westward range extension into the Oriental Region. Based on its male hypopygial morphology, the species clearly falls within the clade characterised by spines on the mesal face of the gonostylus (Kim and Bae 2023). Its occurrence in Yunnan suggests that the species is not restricted to temperate Northeast Asia but extends into subtropical montane forests of south-western China, filling a major geographical gap between the coastal East Asian populations and the montane fauna of the Oriental Region.

#### Dicranomyia (Erostrata) koreana

Taxon classificationAnimaliaDipteraLimoniidae

Kim & Bae, 2023

44F193E7-3015-59E6-A079-E3EAA3A0195B

Figs 4, 5, 6, 7

Dicranomyia (Erostrata) koreana Kim & Bae, 2023: 200. Type locality: South Korea.

##### Specimens examined.

China • 1♂; Henan Province, Xinxiang City, Huixian City, Shangbali Town, Baligou Scenic Area; 35°36'9.35"N, 113°34'9.03"E; 584.9 m a.s.l.; 11 May 2017; Xin Li leg.; CAU • 1♂; Shandong Province, Qingdao City, Chengyang District, Xiazhuang Subdistrict, Qingyu Village; 36°16'27.10"N, 120°31'35.21"E; 21 Jun.–15 Jul. 2022, Malaise trap; QAU • 1♂; Shandong Province, Qingdao City, Chengyang District, Xifu Town Subdistrict, Qijiangu Scenic Area; 36°17'51.54"N, 120°34'13.71"E; 15 Jul.–23 Aug. 2022, Malaise trap; QAU • 3♂♂; Shandong Province, Qingdao City, Laoshan District, Beizhai Subdistrict, Beijiushui Scenic Area; 36°13'27.35"N, 120°34'55.02"E; 17 Jul.–31 Aug. 2022, Malaise trap; QAU • 1♂1♀; Shandong Province, Qingdao City, Laoshan District, Shazikou Subdistrict, Laoshan Jufeng Scenic Area; 36°10'9.93"N, 120°37'27.22"E; 16 Jul.–16 Aug. 2022, Malaise trap; QAU.

**Figure 4.**
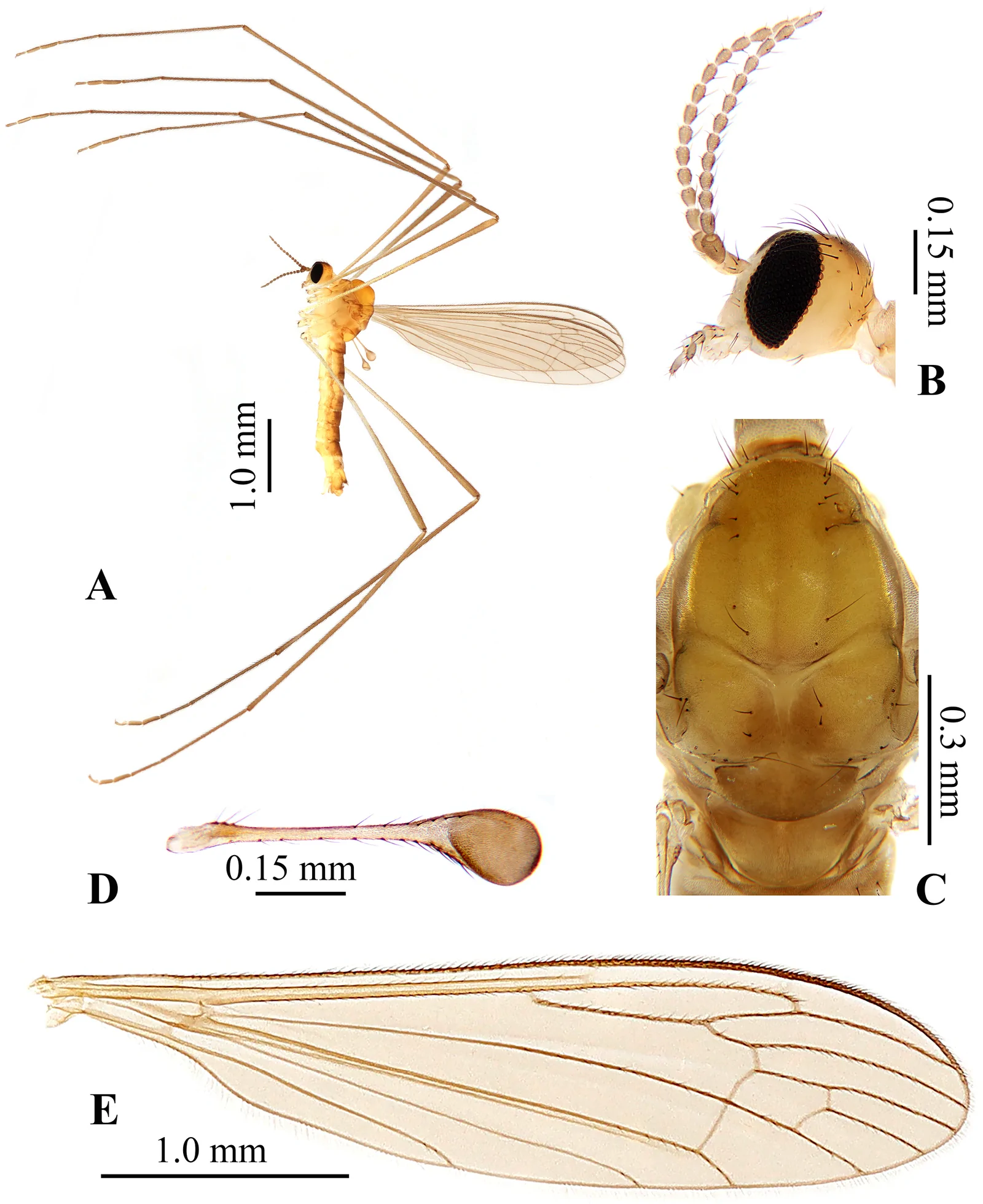
Dicranomyia (Erostrata) koreana Kim & Bae, 2023. **A**. Habitus of male, lateral view; **B**. Head, lateral view; **C**. Thorax, dorsal view; **D**. Halter; **E**. Wing.

**Figure 5.**
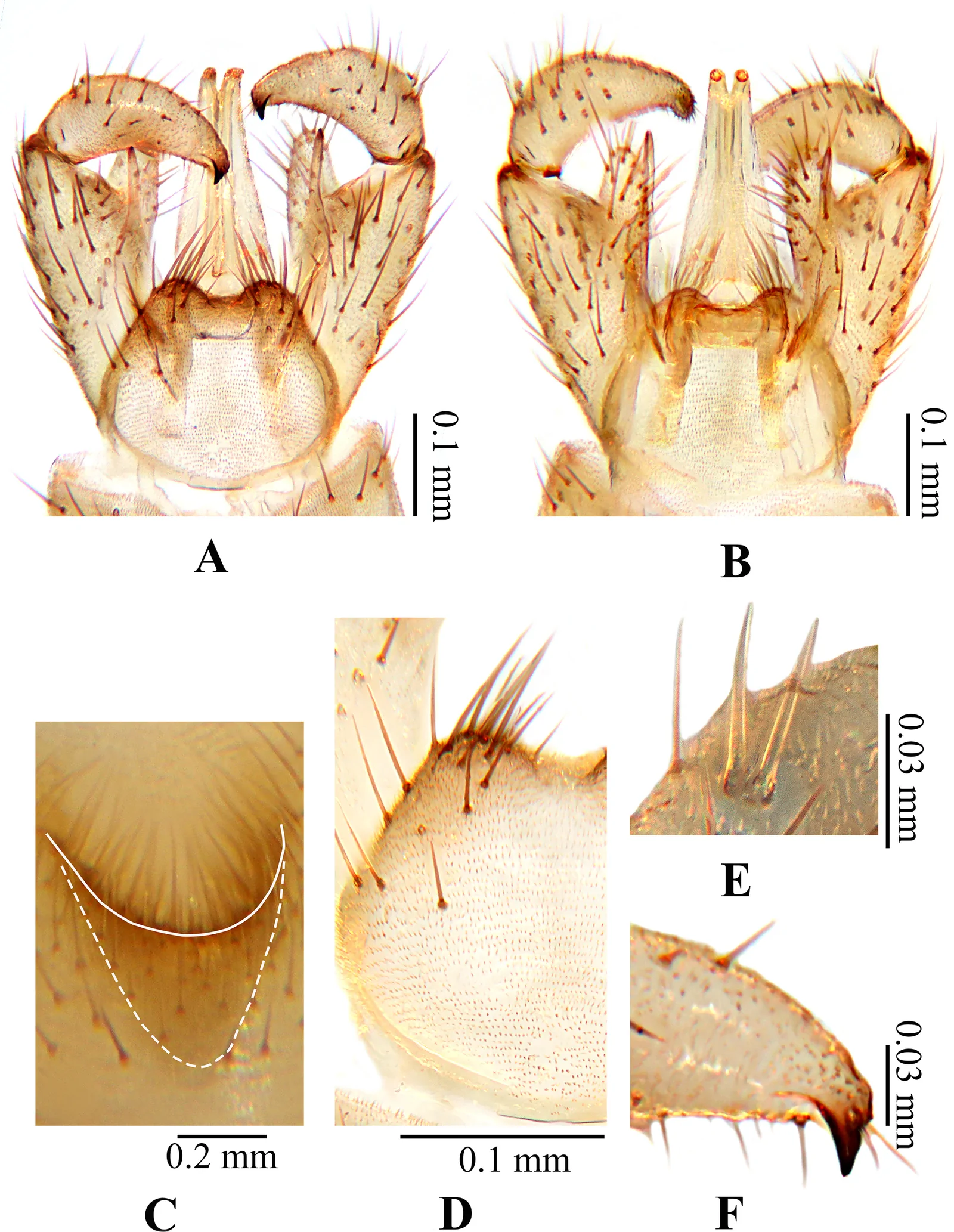
Dicranomyia (Erostrata) koreana Kim & Bae, 2023. **A**. Male hypopygium, dorsal view; **B**. Male hypopygium, ventral view; **C**. Sac of male sternite 7, ventral view (outlined boundary); **D**. Tergite 9, dorsal view; **E**. Basal part of gonostylus, lateral view; **F**. Apical part of gonostylus, dorsal view.

**Figure 6.**
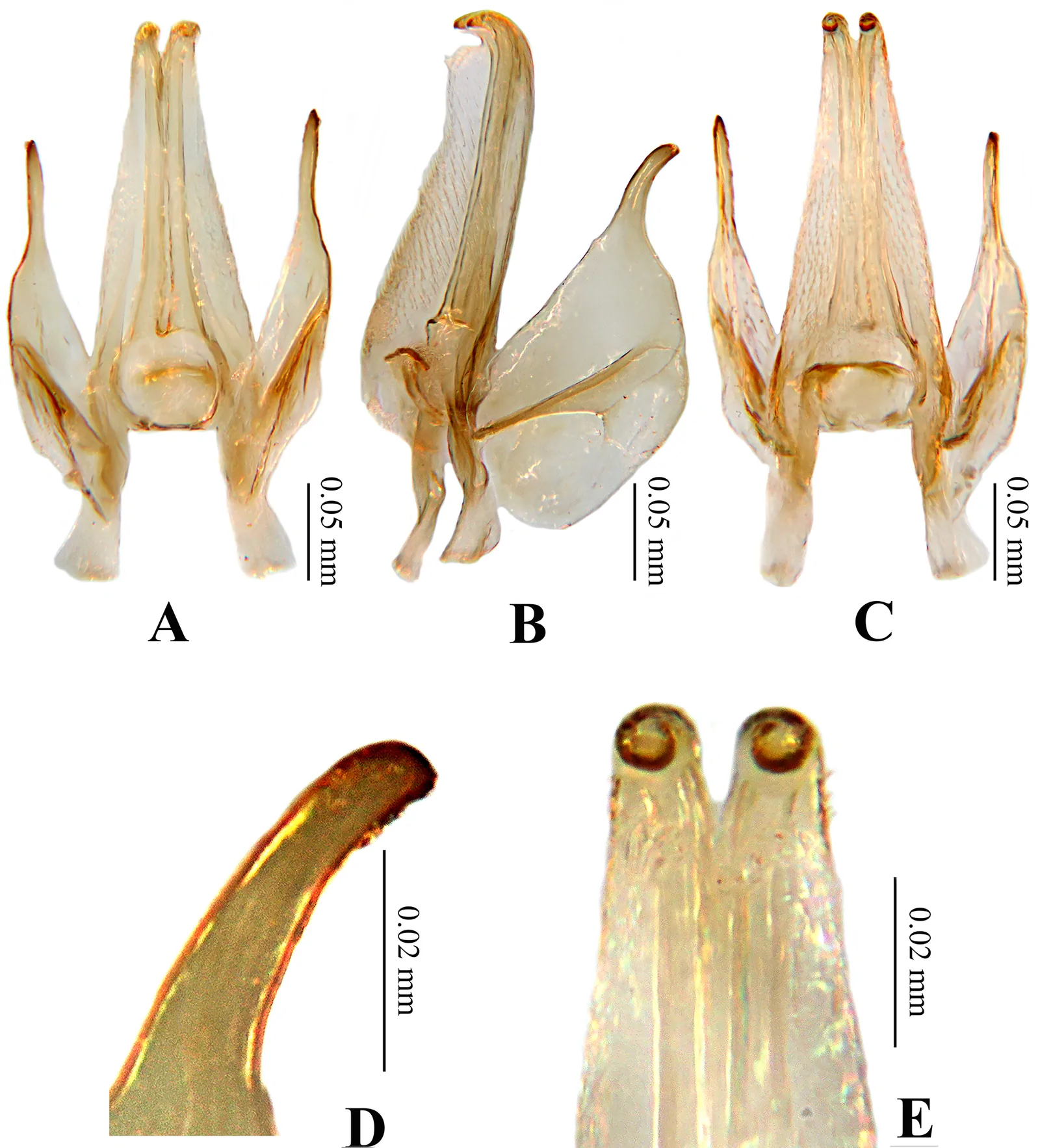
Dicranomyia (Erostrata) koreana Kim & Bae, 2023. **A**. Complex of aedeagus, dorsal view; **B**. Complex of aedeagus, lateral view; **C**. Complex of aedeagus, ventral view; **D**. Apical part of paramere, lateral view; **E**. Apical part of aedeagus, ventral view.

**Figure 7.**
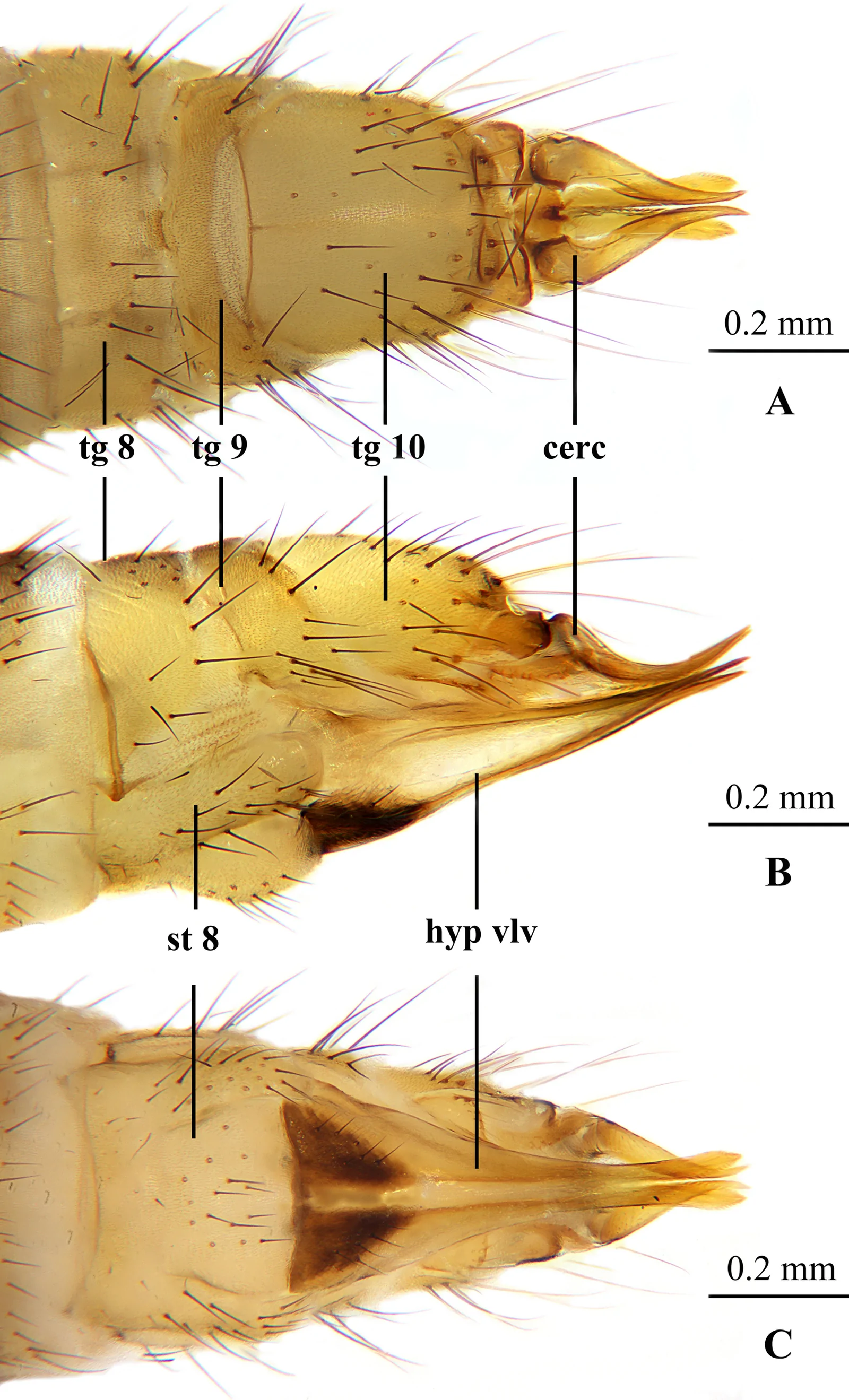
Dicranomyia (Erostrata) koreana Kim & Bae, 2023. **A**. Female ovipositor, dorsal view; **B**. Female ovipositor, lateral view; **C**. Female ovipositor, ventral view. Abbreviations used in figures: cerc = cercus; hyp vlv = hypogynial valve; st = sternite; tg = tergite.

##### Diagnosis.

Prescutum and presutural scutum brownish yellow. Postsutural scutum brown with posteromedial area slightly darker. Pleuron brownish yellow, darker near base of wing. Femora brownish yellow, gradually darkening towards the apex. Vein Sc ending opposite about 1/3 length of Rs, m-cu at base of dm. Tergite 9 posterior margin with two small, rounded lobes separated by U-shaped notch. Gonostylus gradually tapering, tip dorsally with a stout black spine, ventrally with dense short black spines. Paramere with tip rounded and darker. Aedeagus tip forked and recurved.

##### Description.

**Male**. Body length 3.2–4.1 mm, wing length 3.8–5.0 mm.

Head (Fig. 4A, B). Yellow, vertex and occiput pale brown. Setae on head dark brown. Antennae with 14 segments, pale brown with scape paler. Scape cylindrical, 1.5 times as long as wide. Pedicel oval, 1.2 times as long as wide, tip slightly enlarged. Flagellomeres long-oval, outermost flagellomere constricted at tip third, each with 3–5 verticils, outermost one with two additional short setae at tip. Rostrum pale yellow with dark-brown setae. Palpus pale brownish yellow, three segments with dark-brown setae observed.

Thorax (Fig. 4C). Pronotum brownish yellow. Prescutum and presutural scutum brownish yellow. Postsutural scutum brownish yellow with posteromedial area slightly darker. Scutellum dark brownish yellow. Mediotergite brownish yellow with anterior margin darker. Pleuron (Fig. 4A) brownish yellow, darker near base of wing. Thorax with dark brown setae. Coxae (Fig. 4A) pale yellow; trochanters pale yellow; femora brownish yellow, gradually darkening towards apex; tibiae brown; tarsi brown with tip paler. Setae on legs dark brown. Wing (Fig. 4E) brownish yellow; veins brown. Venation: Sc ending opposite about 1/3 length of Rs; sc-r close to tip of Sc; Rs arising from mid-wing, base curved; cell dm closed, 1.5 times as long as wide; m-cu at base of dm. Halter (Fig. 4D) pale brownish yellow, base and knob darker.

Abdomen (Fig. 4A). Abdominal segments 1–7 with tergites brown, sternites brownish yellow, segment 8 pale brownish yellow; sternite 7 (Fig. 5C) with a broad-based internal sac tapering to a slightly pointed apex and bearing a U-shaped opening. Setae on abdomen brown.

Hypopygium (Figs 5, 6). Generally pale brownish yellow. Tergite 9 (Fig. 5A, D) nearly rounded, posterior margin with two small, rounded lobes separated by U-shaped notch, each lobe with about 14–16 long brown setae, and median area also with 1–2 setae. Gonocoxite (Fig. 5A, B) conical, slightly swollen medially; a ventromesal lobe at its middle, with length about 1/3 of gonocoxite. Gonostylus (Fig. 5A, B, E, F) about 2/3 length of gonocoxite, outer side at basal 1/3 with two separate transparent spines (Fig. 5E), gradually tapering, tip dorsally with a stout black spine (Fig. 5F), ventrally with dense short black spines, surface setose. Paramere (Fig. 6A–D) in lateral view broad at base, narrowed and curved apically, with tip rounded and darker. Aedeagus (Fig. 6A–C, E) long, extending to about 1/2 of gonostylus, widened at base, tip forked and recurved.

**Female**. Body length 4.0 mm, wing length 4.2 mm. Generally similar to male in body coloration. Ovipositor (Fig. 7) with tergite 8 brownish yellow, about 1.3 times as long as tergite 9. Tergite 9 brownish yellow. Tergite 10 brownish yellow, about 3.0 times as long as tergite 9. Cercus short, brownish yellow, with tip acute, curved upward. Hypogynial valve brownish yellow, with base brownish black and middle paler, extending to just before tip of cercus.

##### Distribution.

China (Henan, Shandong); South Korea.

##### Remarks.

Dicranomyia (E.) koreana was described very recently from South Korea (Kim and Bae 2023). Its discovery in Shandong and Henan provinces represents the first confirmed occurrence of the subgenus *D.* (*Erostrata*) in the Palaearctic part of China. This extends the species’ known range by more than 1000 km westward, crossing the Yellow Sea. In the molecular phylogeny presented by Kim and Bae (2023), D. (E.) koreana belongs to the clade that lacks strong black spines on the mesal face of gonostylus. Together with D. (E.) tabashii and D. (E.) yazuensis (both also known from Korea and Japan), it forms a lineage that appears to be successful in dispersing across the marginal seas of East Asia. The occurrence of D. (E.) koreana in Henan, well inland from the coast, indicates that the species is not restricted to coastal hills but has penetrated the interior warm-temperate mountains of the North China Plain.

#### Dicranomyia (Erostrata) serrata

Taxon classificationAnimaliaDipteraLimoniidae

Dai & Zhang sp. nov.

E658760A-A35D-5D18-B967-E5FFF9405DA9

https://zoobank.org/2CC6EA89-B9FD-4A5B-A288-5C7CA35DA68F

Figs 8, 9, 10

##### Type material.

***Holotype***: China • 1♂; Sichuan Province, Chongzhou City, Jiguanshan Township, Shaoyaogou Protection Station; 30°45'47.56"N, 103°13'56.62"E; 1400 m a.s.l.; 29 Jul. 2016; Yuqiang Xi leg.; CAU.

**Figure 8.**
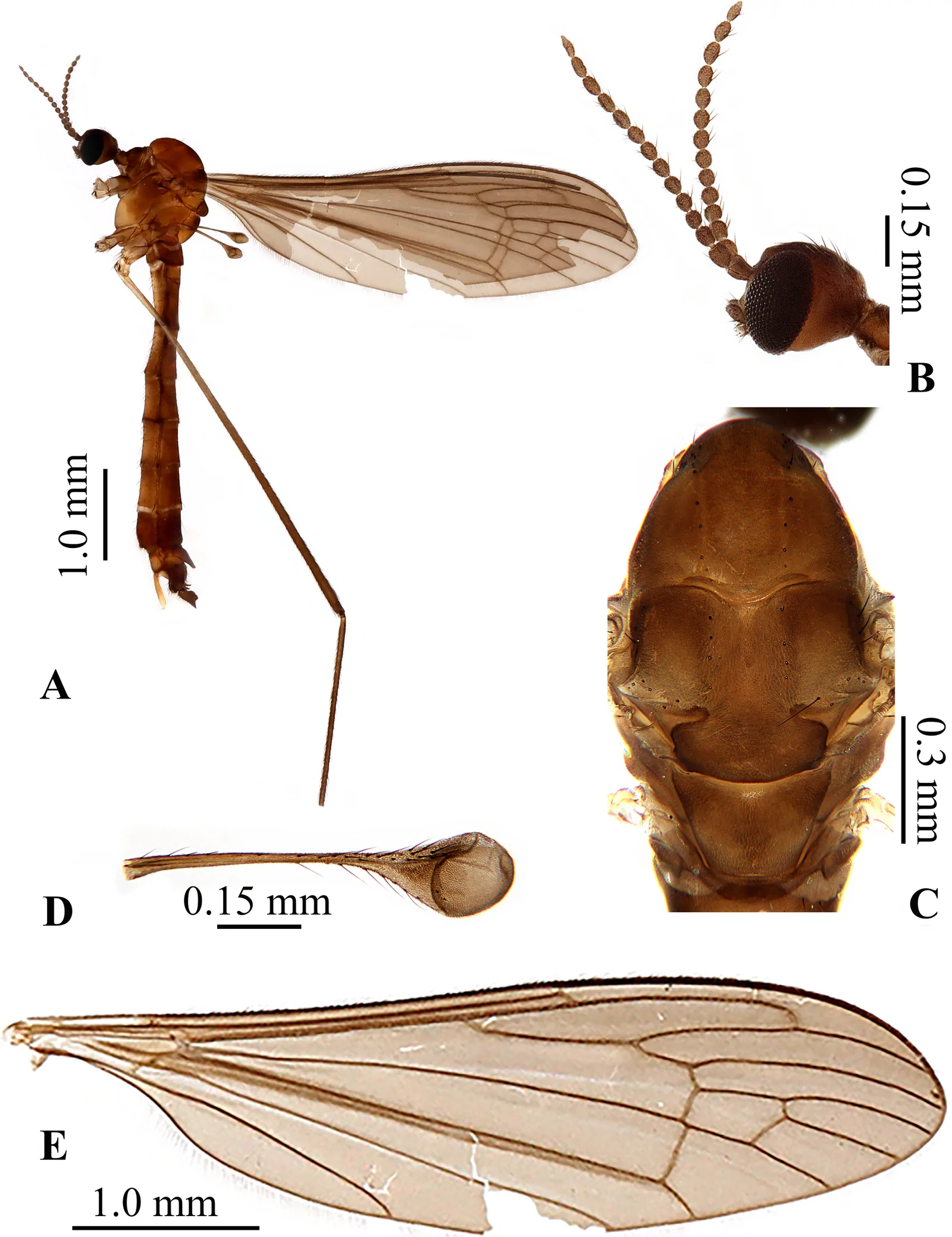
Dicranomyia (Erostrata) serrata Dai & Zhang, sp. nov. **A**. Habitus of male, lateral view; **B**. Head, lateral view; **C**. Thorax, dorsal view; **D**. Halter; **E**. Wing.

**Figure 9.**
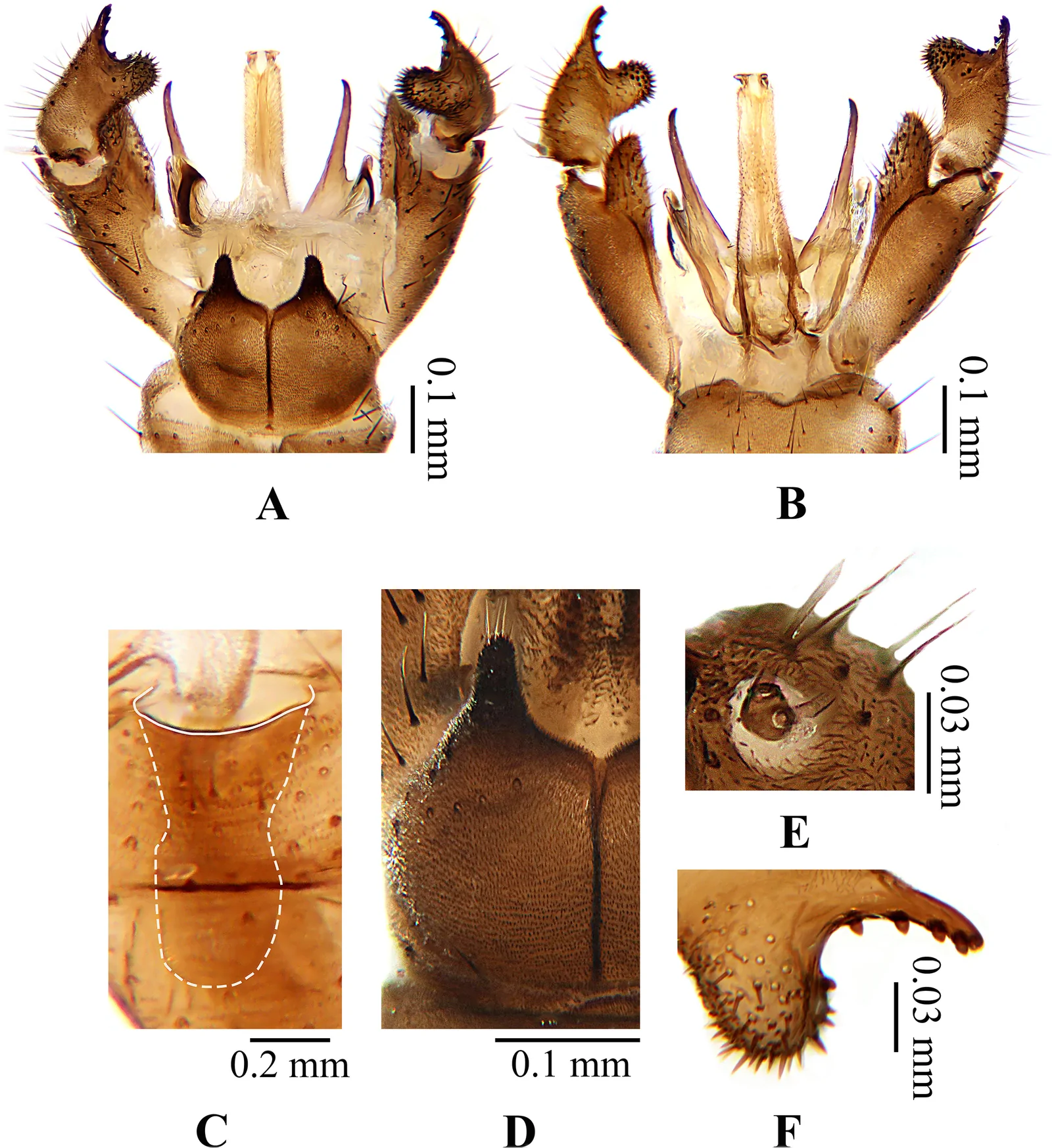
Dicranomyia (Erostrata) serrata Dai & Zhang, sp. nov. **A**. Male hypopygium, dorsal view; **B**. Male hypopygium, ventral view; **C**. Sac of male sternite 7, ventral view (outlined boundary); **D**. Tergite 9, dorsal view; **E**. Basal part of gonostylus, lateral view; **F**. Apical part of gonostylus, dorsal view.

**Figure 10.**
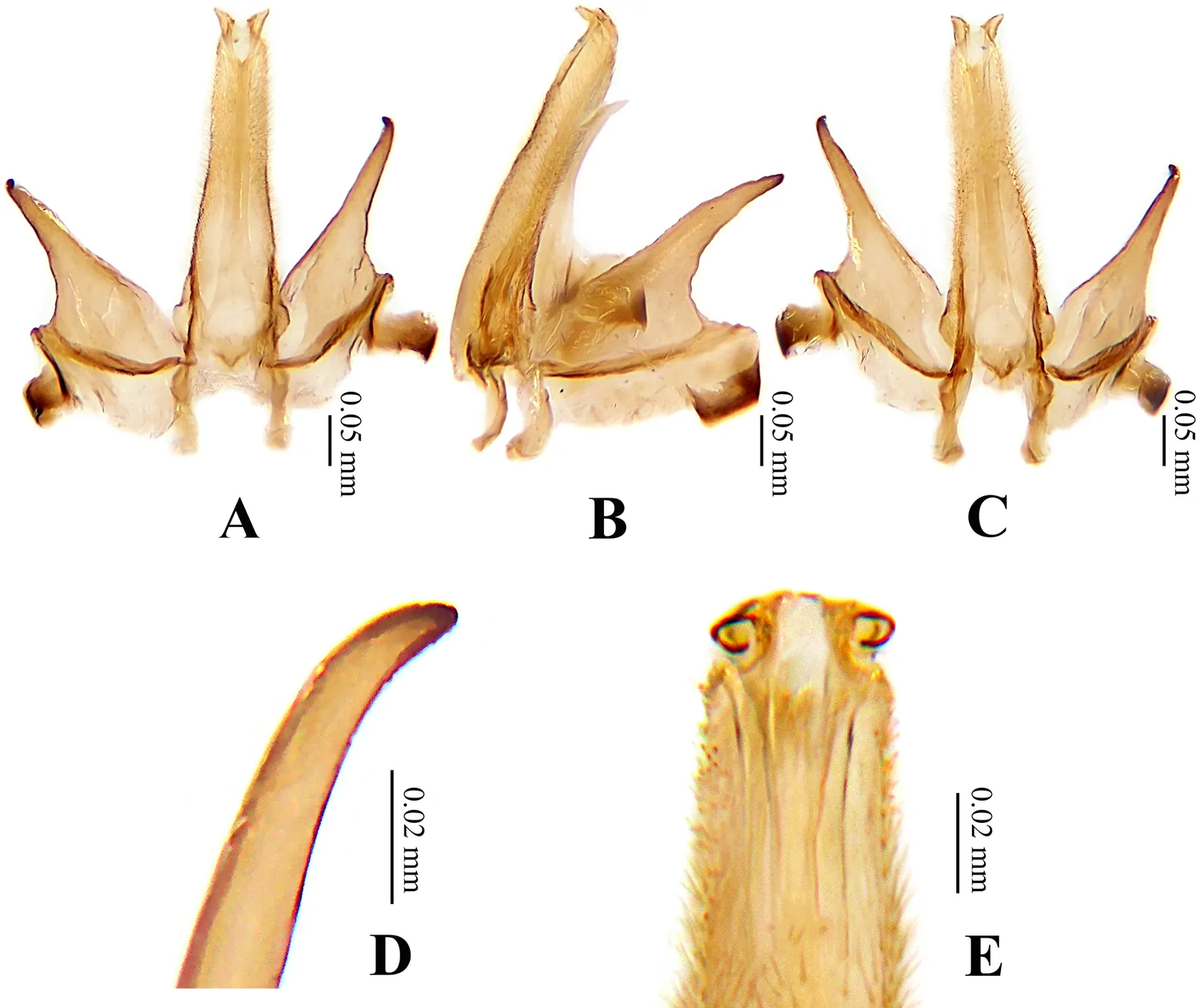
Dicranomyia (Erostrata) serrata Dai & Zhang, sp. nov. **A**. Complex of aedeagus, dorsal view; **B**. Complex of aedeagus, lateral view; **C**. Complex of aedeagus, ventral view; **D**. Apical part of paramere, lateral view; **E**. Apical part of aedeagus, ventral view.

##### Diagnosis.

Prescutum and presutural scutum brown with anterior 1/3 margin slightly darker. Postsutural scutum brown with median area slightly paler. Pleuron brown, darker near base of wing. Wing pale brown, with darker areas on upper 1/2 (veins Sc, Rs, R_1_–R_4_). Vein Sc ending opposite about 2/3 length of Rs, m-cu just beyond base of dm. Tergite 9 posterior margin with a broad, U-shaped notch, forming two elongate projections. Gonostylus tip distinctly concave, forming a thin upper arm with 4–5 teeth and a circular lower arm densely covered with short, black spines. Paramere tip subacute, with extreme tip bending inward abruptly. Aedeagus tip forked and recurved, with subtip constricted.

##### Description.

**Male**. Body length 5.3 mm, wing length 5.0 mm.

Head (Fig. 8A, B). Brown, vertex and occiput darker. Setae on head dark brown. Antennae with 14 segments, brown. Scape cylindrical, 1.5 times as long as wide. Pedicel oval, 1.5 times as long as wide, tip slightly enlarged. Flagellomeres long-oval, outermost flagellomere slightly elongate and tapering, each with 3–4 verticils. Rostrum brown with dark-brown setae. Palpus dark brown, one segment with dark-brown setae observed (additional segments likely absent due to specimen condition).

Thorax (Fig. 8C). Pronotum brown. Prescutum and presutural scutum brown with anterior 1/3 margin slightly darker. Postsutural scutum brown with median area slightly paler. Scutellum dark brown. Mediotergite brown with anterior margin slightly darker. Pleuron (Fig. 8A) brown, ventral side of katepisternum slightly paler. Thorax with dark-brown setae. Coxae (Fig. 8A) pale brown; trochanters slightly paler in comparison to coxae; femora brown, with base slightly paler; tibiae brown. Setae on legs dark brown. Wing (Fig. 8E) pale brown, with weakly darker areas on upper 1/2 (veins Sc, Rs, R_1_–R_4_); veins brown. Venation: Sc ending opposite about 2/3 length of Rs; sc-r very close to tip of Sc; Rs arising from middle of wing, base slightly curved, nearly straight; cell dm closed, 2.0 times as long as wide; m-cu just beyond base of dm. Halter (Fig. 8D) brown, knob paler.

Abdomen (Fig. 8A). Segments 1–8 brown, with segments 1 and 8 darker; sternite 7 (Fig. 9C) with an internal sac, broad basally, slightly narrowed towards apex, constricted medially, and bluntly rounded at the tip, bearing a broadly U-shaped opening. Setae on abdomen dark brown.

Hypopygium (Figs 9, 10). Generally brown, tip of tergite 9 darker. Tergite 9 (Fig. 9A, D) nearly trapeziform, medially with longitudinal line, posterior margin with a broadly U-shaped notch, forming two elongate blackened projections. Each projection with 4–5 setae at tip, each side of tergite 9 also with 6–8 setae. Gonocoxite (Fig. 9A, B) in dorsal view cylindrical, tip slightly enlarged, inner side concave at midlength, and in ventral view fusiform, inner side slightly convex; a ventromesal lobe at its apical third, with tip slightly acute, length about 1/2 of gonocoxite. Gonostylus (Fig. 9A, B, E, F) length about 1/2 of gonocoxite, outer side at base with two long, stout, transparent setae (Fig. 9E); tip distinctly concave, forming a thin upper arm with 4–5 teeth and a circular lower arm densely covered with short, black spines (Fig. 9F). Paramere (Fig. 10A–D) in lateral view broad at base, narrowed and nearly straight apically, tip subacute; with extreme tip bending inward abruptly in dorsal view. Aedeagus (Fig. 10A–C, E) long, extending to about tip of gonostylus, widened at base, tip forked and recurved; subtip constricted.

**Female**. Unknown.

##### Etymology.

The specific epithet is a Latin adjective serrata (meaning “serrated”). It refers to the four or five teeth arranged in a serrated pattern on the upper arm of the gonostylus.

##### Distribution.

China (Sichuan).

##### Remarks.

The new species is placed in *D.* (*Erostrata*) based on the reduction of the rostrum, the wing venation (vein Sc ending at about 2/3 the length of Rs) and the male hypopygial characters (possessing only one pair of gonostyli). Its gonostylus bears strong black spines on the inner side, which places it within the “spinose” clade of Kim and Bae (2023). The new species is most similar to D. (E.) melas, D. (E.) globithorax, and D. (E.) globulithorax, but it can be distinguished from all three by the posterior margin of tergite 9, which has a broadly U-shaped notch forming two elongate projections and the gonostylus tip distinctly concave, forming a long spine and a circular protuberance densely covered with black spines. In addition, it differs from D. (E.) melas in having a paramere with a less strongly curved apex and lacking a subapical black point; from D. (E.) globithorax in having a less gibbous mesothorax and the different shape of the internal sac of sternite 7; and from D. (E.) globulithorax in having a more slender aedeagus and a paramere with a less acute apex. The type locality, situated on the eastern edge of the Hengduan Mountains in Sichuan, is the farthest inland record for the subgenus. It fills a crucial gap between the known Oriental Region species of the Himalayas and the coastal East Asian species.

### Geographical distribution

**Figure 11.**
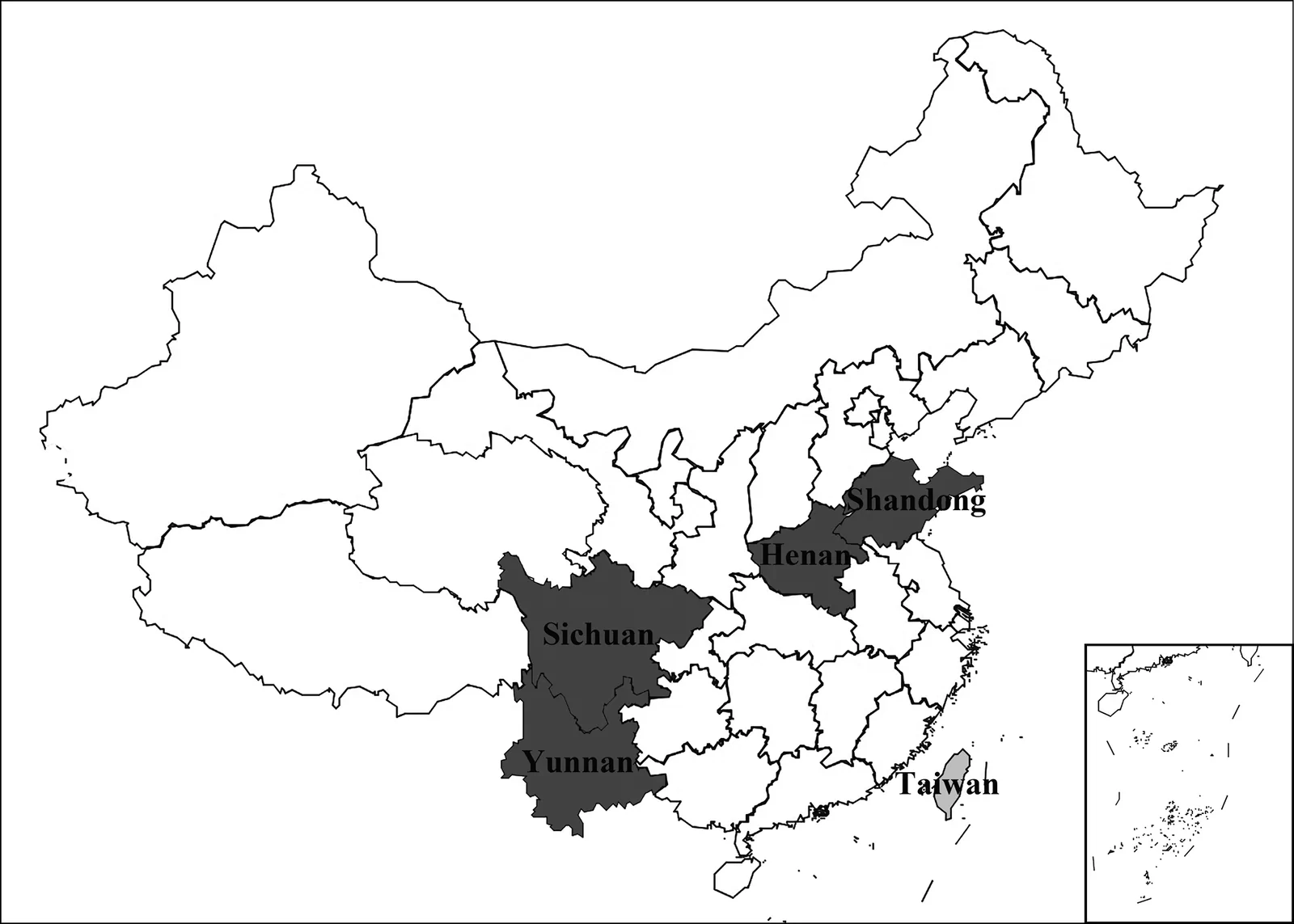
Distribution map of Dicranomyia (Erostrata) species in China (light grey area: previously known distribution record; dark grey areas: new distribution records in this study).

Prior to this study, only one species, D. (E.) melas, was recorded from China, at a locality in Taiwan. Our findings bring the number of confirmed Chinese species to four, distributed across at least five provinces (Fig. 11). The Chinese distribution shows a clear bipartite pattern divided by the Qinling–Huaihe Line: a northern Palaearctic component represented by D. (E.) koreana in warm-temperate stream forests of Shandong and Henan and a southern Oriental component composed of the new species in subtropical montane forests of Sichuan. Notably, D. (E.) globulithorax, newly recorded from Yunnan, bridges the two regions, as it is primarily a Palaearctic species that extends south-westward into the Oriental part of China. Thus, China is unique in hosting representatives of both zoogeographical regions well inland from the coast. The discovery of D. (E.) koreana in Shandong and Henan represents the first confirmed occurrence of the subgenus in the Palaearctic part of China. The new records can be explained by Pleistocene sea-level fluctuations: during glacial maxima, the Yellow Sea basin was largely exposed, connecting the Korean and Shandong peninsulas. The present disjunct distribution of D. (E.) koreana is likely a relict of this formerly continuous range. The Henan populations further suggest that the species was able to spread inland along the forested margins of the lower Yellow River valley. This pattern encourages targeted surveys for other Korean–Japanese Palaearctic species in the mountains of northern China. On the Oriental side, the new data profoundly reshape the understanding of the group’s distribution. D. (E.) globulithorax, previously known from Japan, Russian Far East and South Korea, is here reported from Yunnan, but no populations have been found in the intervening provinces of northern or north-eastern China. The Yunnan population thus represents the southernmost known record of the species, isolated by approximately 2000 km from its previously known range. The Yunnan population of D. (E.) globulithorax, the new Sichuan species and D. (E.) melas are situated at almost the same latitude as the Himalayan D. (E.) cnephosa and D. (E.) congesta. Geographically, this links the four previously disconnected distribution centres—the southern Himalayas, the Hengduan Mountains, the Yunnan-Guizhou Plateau, and Taiwan—into a single chain. We therefore propose the “Southern Mountain Corridor” hypothesis, which posits that the Oriental *D.* (*Erostrata*) were able to disperse along a nearly continuous belt of moist subtropical montane forest. The likely corridor runs from northern Myanmar through western Yunnan and Sichuan, via the Qinling–Daba Mountains to western Hubei, Guizhou, western Hunan, the Nanling and Wuyi Mountains, and ultimately to Taiwan. The new Sichuan species, situated at the pivotal junction of this corridor on the eastern edge of the Hengduan Mountains, provides the strongest direct evidence yet for this biogeographical connection.

Worldwide, the subgenus *Erostrata* currently comprises 14 species (excluding the new species described here). In the Nearctic Region, only D. (E.) globithorax occurs, ranging across North America; it is also widespread in the Palaearctic Region (Japan, South Korea), making it the sole species exhibiting a trans-Beringian disjunction and thus of exceptional historical biogeographical significance. All eight Palaearctic species are confined to the East Asian margin: D. (E.) globithorax, D. (E.) globulithorax, D. (E.) jejuensis, D. (E.) koreana, D. (E.) reniformis, D. (E.) submelas, D. (E.) tabashii, and D. (E.) yazuensis. All eight occur in Japan and/or South Korea, establishing the Japan–Korea–Russian Far East arc as the indisputable centre of Palaearctic diversity. The Chinese record of D. (E.) koreana represents a westward extension of this core. In addition, the first record of D. (E.) globulithorax from China (Yunnan) represents a significant south-westward extension of this otherwise Palaearctic species into the Oriental Region. Together with the new species from Sichuan, the Oriental species now show a more continuous belt along the southern Himalayas and the mountains of south-western China. Placed in this global context, China (with four species) emerges as another centre of diversity for the subgenus, second only to Japan (six species) and Korea (seven species) in species richness, with its true diversity certainly higher given the extensive undersurveyed areas in central and northern China. A three-tiered global pattern is established: (i) the coastal East Asian core (Japan, Russian Far East, South Korea), with the highest species richness and endemism; (ii) the emerging inland Chinese centre (Henan, Shandong, Sichuan, Yunnan), which hosts representatives of both major clades and extends their ranges deep into the continent; and (iii) the peripheral dispersal fringes (India, Indonesia, Malaysia, Nepal, Philippines) (Table 1). China is the geographic hub linking the eastern and western, and northern and southern components of the subgenus’ range. Through its warm-temperate North China Plain and its subtropical south-western mountains, connects the Palaearctic core with the Oriental periphery, making it the key region for understanding the evolutionary and biogeographical history of *D.* (*Erostrata*) in East Asia.

**Table 1.** Species of Dicranomyia (Erostrata) grouped by the three global distributional tiers.

**Tier**	**Species**	**Zoogeographical region**	Distribution*
Coastal East Asian core	D. (E.) globithorax	Nearctic/Palaearctic	Canada, Japan, South Korea, USA
	D. (E.) globulithorax	Palaearctic/Oriental	**China (Yunnan)**, Japan, Russian Far East, South Korea
	D. (E.) jejuensis	Palaearctic	South Korea
	D. (E.) koreana	Palaearctic	**China (Henan, Shandong)**, South Korea
	D. (E.) reniformis	Palaearctic	Japan
	D. (E.) submelas	Palaearctic	Japan, South Korea
	D. (E.) tabashii	Palaearctic	Japan, North Korea, Russian Far East, South Korea
	D. (E.) yazuensis	Palaearctic	Japan, South Korea
Emerging inland Chinese centre	D. (E.) melas	Oriental	China
	D. (E.) serrata sp. nov.	Oriental	**China (Sichuan)**
Peripheral dispersal fringes	D. (E.) canis	Oriental	Philippines
	D. (E.) cnephosa	Oriental	Nepal
	D. (E.) congesta	Oriental	India
	D. (E.) cynotis	Oriental	Philippines
	D. (E.) striopleura	Oriental	Indonesia, Malaysia

*Bold entries highlight findings of the present study. Dicranomyia (E.) globulithorax and D. (E.) koreana are listed in the Coastal East Asian core based on their known ranges, but their first records from China in this study also contribute to the Emerging inland Chinese centre.

## Discussion

This study increases the number of *D.* (*Erostrata*) species confirmed from China from one to four and provides the first key for their identification. Kim and Bae (2023) showed that *D.* (*Erostrata*) comprises two deeply divergent genetic lineages that correlate perfectly with the presence or absence of strong black spines on the mesal face of the gonostylus. The new species from Sichuan offers a critical test of this morphological–molecular framework. Although molecular data are not yet available for our Chinese material, the new species possesses a spinose mesal face of the gonostylus and, thus, can be placed morphologically within the spinose clade together with D. (E.) globulithorax. This clade comprises both coastal insular and deep inland members and the new species extends the known inland range of this clade. This finding adds an important data point to the growing understanding of the subgenus’ diversification. Future *COI* barcoding of these Chinese samples will allow quantitative genetic distance comparisons and further test the stability of the subgeneric concepts.

The two major findings of this study—the trans-Yellow Sea disjunction and the mainland occurrence of D. (E.) globulithorax in Yunnan together with the Sichuan new species—support a dual dispersal model for *D.* (*Erostrata*) into continental East Asia. The Palaearctic lineage(s) likely expanded along a northern warm-temperate corridor during Pleistocene low sea-level stands, whereas the Oriental lineage(s) dispersed along a southern subtropical mountain corridor, potentially much earlier. The Yunnan record of D. (E.) globulithorax, a primarily Palaearctic species extending southward into the Oriental Region, provides supporting evidence of a northern element penetrating the southern mountain corridor. The new Sichuan species, situated at the junction of the Hengduan Mountains and the Sichuan Basin, represents the most compelling single piece of evidence for the Southern Mountain Corridor hypothesis.

With the discovery of the first mainland Chinese populations, the distribution map of *D.* (*Erostrata*) has entered a new phase. Priority areas for future surveys include the mountains of northern China for additional Palaearctic species, the central Chinese mountains to test the continuity of the Southern Mountain Corridor, and the south-eastern Chinese mountains, which may help clarify the biogeographical connection between Taiwan and the mainland Oriental fauna. Such surveys will undoubtedly yield further new records and likely additional new species, consolidating China’s position as an emerging major inland centre of *D.* (*Erostrata*) diversity.

## Supplementary Material

XML Treatment for Dicranomyia (Erostrata) globulithorax

XML Treatment for Dicranomyia (Erostrata) koreana

XML Treatment for Dicranomyia (Erostrata) serrata

## Additional information

### Artificial Intelligence (AI) use

The authors accept full responsibility for the content of the manuscript, including the disclosure of any use of AI.

No AI tools were used in the preparation of this manuscript.

### Author ORCIDs

L. Dai https://orcid.org/0009-0008-9525-9942

J. Chen https://orcid.org/0009-0001-0049-5192

S. Ma https://orcid.org/0009-0006-8115-6526

P. Guo https://orcid.org/0009-0007-3912-1802

X. Zhang https://orcid.org/0000-0002-8054-0968

### Data availability

All of the data that support the findings of this study are available in the main text.
